# Is virtual reality suitable for hand hygiene training in health care workers? Evaluating an application for acceptability and effectiveness

**DOI:** 10.1186/s13756-022-01127-6

**Published:** 2022-06-25

**Authors:** Vanessa M. Eichel, Christian Brandt, Juliane Brandt, Jonas M. Jabs, Nico T. Mutters

**Affiliations:** 1 Section for Hospital Hygiene and Environmental Health, Center for Infectious Diseases, Heidelberg University Hospital, Im Neuenheimer Feld 324, 69120 Heidelberg, Germany; 2 Department of Hematology, Oncology and Rheumatology, Heidelberg University Hospital, Im Neuenheimer Feld 410, 69120 Heidelberg, Germany; 3 Hospital of Barmherzige Brüder Tier, Internal Medicine 1 Infectiology, Nordallee 1, 54292 Trier, Germany; 4 Institute for Hygiene and Public Health, University Hospital Bonn, Venusberg-Campus 1, 53127 Bonn, Germany

## Abstract

**Background:**

For effective prevention of nosocomial transmissions continuous training and motivation of health care workers (HCW) are essential to maintain and increase compliance with high rates of hand hygiene. The use of Virtual Reality (VR) seems to be a contemporary and interesting approach for hand hygiene training in HCW. Nevertheless, HCW should be asked for their preferences as intrinsic motivation is essential for compliance with hand hygiene and training success should be evaluated.

**Methods:**

A prospective, cross-controlled trial was conducted at two wards in a tertiary care hospital comparing a conventional lecture for hand hygiene to the use of VR. Both interventions were assigned at ward level. Primary outcome was HCW acceptance, which was verified in a third ward, secondary outcomes were hand rub consumption and compliance to indications for hand hygiene as proposed by WHO.

**Results:**

In summary, 81 trainings were conducted, 48 VR trainings and 33 trainings by lecture. VR training was well accepted by HCW with a mean score in all items from 3.9 to 4.3 (out of 5). While most HCW (69%) would prefer VR teaching rather than a lecture for hand hygiene education, only 4% preferred the traditional lecture. 400 observations of hand hygiene indications were made, 50 before intervention and 50 after each intervention at the three wards. Mean proportion of correct and indication-appropriate performances was 81% before intervention, 87% after VR training (*p* = 0.12), and 95% after lecture (*p* = 0.04). Hand rub consumption did not change significantly in any group.

**Conclusions:**

Due to the high acceptance of VR technology among healthcare workers, it can be considered an interesting addition to conventional lectures for teaching hand hygiene. However, the hypothesis that VR teaching has a higher impact on hand rub use and hand hygiene compliance than a conventional lecture cannot be confirmed.

## Introduction

Healthcare-associated infections (HAI) are the most frequent adverse event in health care facilities and it is assumed that about one third could be prevented [1, 2]. In addition to the immense health risk for patients, this places a massive financial burden on the health care system. In the United States for example, costs of annually $4.5 billion have been estimated in this context [3]. Though, adequate hand hygiene can significantly reduce health-care associated infections and the World Health Organization (WHO) states that an improvement in hand hygiene is a key factor in reducing global health care associated infections [4]. However, studies have shown poor compliance with hand hygiene in HCW with an overall compliance rate of about 40% [1]. Therefore, continuous training and motivation of health care workers (HCW) are essential to maintain and increase compliance rates. Well established methods are group and individual trainings, practice trainings, reminders, observation and feedback, role-plays, competitions, reward systems, online courses, posters, and distribution of information materials.

Recently, simulation methods are increasingly used to specifically train HCW, as they offer a broad spectrum of trainable situations and may facilitate the transfer of theoretical knowledge into clinical practice [5–7]. Within the simulation, the user has the opportunity to live an artificial but realistic experience and boost his power of imagination [8, 9]. Among these methods, VR is an emerging technology that offers multiple benefits [7]. Training can be repeated as many times as needed, doesn’t require an instructor or trainer and can thus be performed 24/7. Also, the simulation can be adapted to the learners level of knowledge and the training is tailored to the learner and creates a true-to-life experience [10]. Over the last two decades a wide variety of medical applications have been identified [11]. In clinical context, such as the treatment and the diagnosis of psychiatric disorders, VR training methodology has already proven to be a useful approach [12]. Thus, VR seems to be a contemporary and interesting approach for hand hygiene training in HCW. Nevertheless, HCW should be asked for their preferences as intrinsic motivation is essential for compliance with hand hygiene and training success should be evaluated.

Therefore, this study compares VR technology with a conventional lecture in terms of user acceptance and satisfaction and effectiveness in hand hygiene training of HCW.

## Methods

The study was carried out with HCW of three wards in a tertiary care hospital in Germany. The investigation period was from 01/April/2020 to 31/March/2021.

### Study design

This interventional study was performed in a prospective, cross-controlled trial design. CRe-DEPTH criteria for describing and evaluating training interventions in healthcare professions are provided in Table 1 [13].

**Table 1:** CRe-DEPTH criteria

Item	Description
1. Aim or objectives of the training	The aim of this study was to compare the VR technology with a conventional lecture in terms of user acceptance and clinical outcome towards hand hygiene
2. Underlying theoretical framework	Nosocomial infections pose an enormous threat to patient safety. Poor hand hygiene is one of the key factors in the spread of germ in healthcare and continuous training is one of the most effective measures in improving the adherence. VR offers a new approach in training by giving an individual, fun, and true-to-life experience as well as offering the opportunity of training 24/7
3. Developmental process	The VR hand hygiene scenario was developed and provided by the company Essity. The compared lecture was closely related to the VR training in content and duration of the lesson
4. Target population and setting of the training	Target population was health-care workers of 3 wards at a tertiary care hospital in Germany
5. Educational resources	We used 4 VR headsets with the installed VR application, a teaching room with 4 swivel chairs and a technical instructor. The lecture was hold as a classic slide show presentation by beamer in a fully seated room
6. Content of the intervention	*VR training*: Initially a short explanation of the correct technique of hand hygiene is given by means of illustrations. After choosing whether the participants profession is nurse or doctor, the first out of three virtual patient rooms can be entered. Finally, various situations take place in which tasks relating to the topic of hand hygiene are to be solved. In particular, the participant will have to decide in different clinical situations whether hand hygiene or the wearing of gloves is necessary, as well as to choose the correct sequence of these. The program immediately gives an alarm if an indication has been forgotten. After completion of the scenarios, the app directly evaluates the situations and gives feedback regarding the correct application of hand hygiene and glove use *Lecture*: A slide show presentation with the correct technique, indications for hand hygiene, and practical cases similar to those in the VR scenario was presented
7. Format	To assess satisfaction and effectiveness of both educational methods, we compared the different approaches in 3 wards. In a two-month interval both trainings were offered in ward 1 and 2 in a crossover design. While ward 1 received the VR training first, ward 2 started with the lecture To increase number of participants a further ward received VR training. Both, the VR scenario and the lecture had a training duration of about 20 min
8. Didactic methods of training	Simulation of scenarios by VR or lecture with slide show were applied
9. Tailoring of the training	In the VR intervention groups, the training can be tailored to profession and speed. The generation of a result overview also provides individual feedback at the end of the training. The lecture groups received all the identical lesson, no differences were made between professions
10. Providers of the training	The introduction and technical support of the VR training was provided by IPC physicians and technical staff. Lecture was provided by IPC physicians
11. Measured outcomes	Primary outcome was the HCW satisfaction. Secondary outcomes were hand rub consumption and compliance to indications for hand hygiene as proposed by WHO [16]
12. Applied assessment method, including its validity and reliability	HCW satisfaction was measured after intervention using a standardized questionnaire with Likert-Scale. Hand rub consumption was measured continuously. Compliance observations were made before and after each intervention. To exclude acute and only short-lived behavioral adjustments compliance was measured after approximately 2-weeks post intervention. It was performed by a research assistant not included in further processes of the trial to guarantee blinding

Two dates were set for the interventions on ward 1 and 2: At the first intervention date, the VR training was held at ward 1, while a face-to-face lecture on the same topic took place at ward 2. After a washout phase of 3 months, the respective training was carried out in the reverse setting. Furthermore, an additional VR training was performed at ward 3 to increase the number of VR participants.

Primary outcome was HCW satisfaction and secondary outcomes were hand rub consumption and compliance to indications for hand hygiene as proposed by WHO measured by observations. For the participants’ safety, adverse effects of the VR glasses training were monitored. Both interventions, the lecture and the VR training, took approximately 20 min to complete.

### VR training

**Fig. 1:**
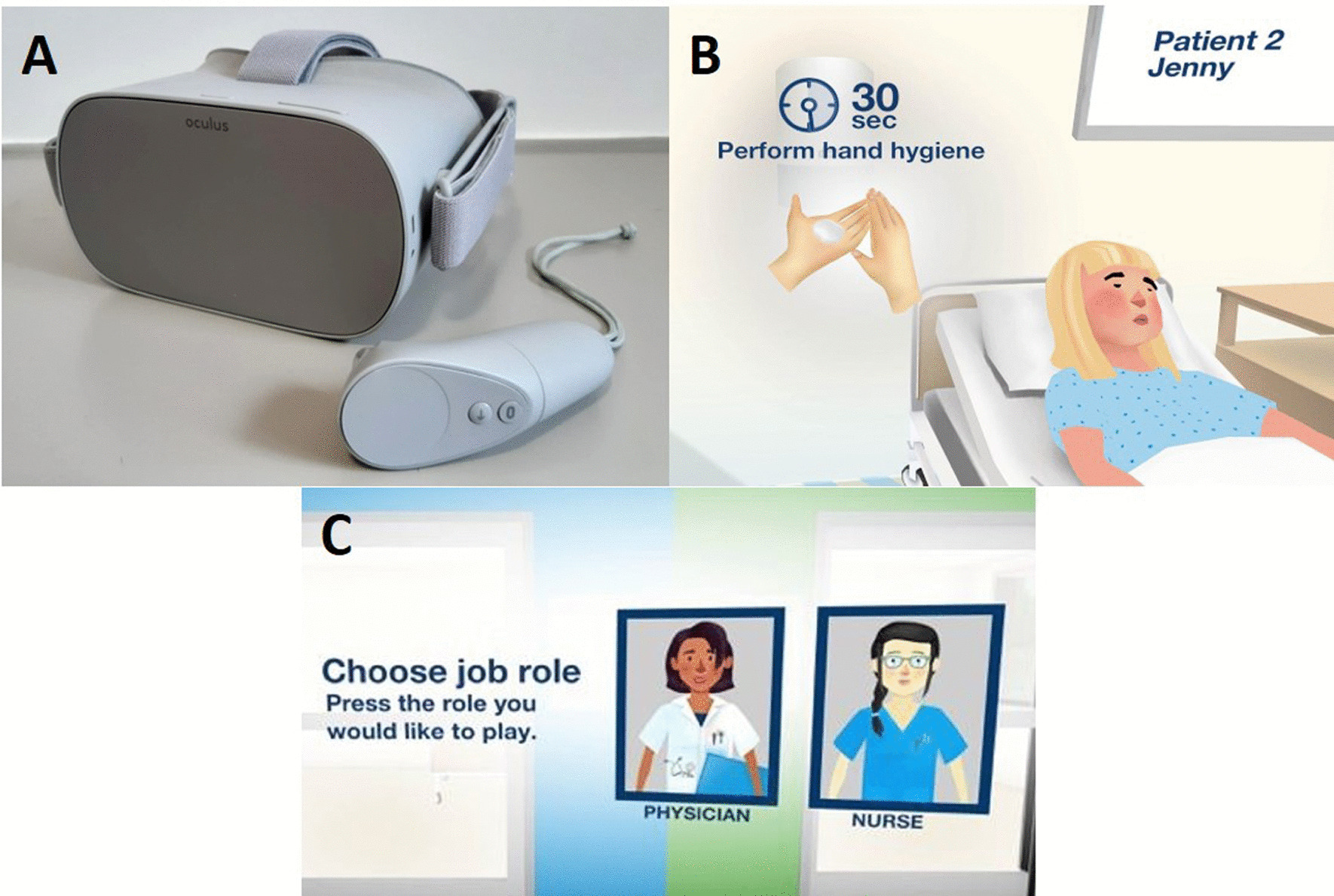
VR Training hard- and software. **A** Oculus go headset and controller; **B** Screenshot during the lesson; courtesy of Essity. **C** Selection of profession; courtesy of Essity

After detailed explanation of the project and time for reflection, the included participants signed a declaration of consent. Every participant received a brief introduction to the use of the VR headset (Oculus go, Meta) by the study team and started the application “VR Clean Hands” (marketed by Essity Health and Hygiene AB, Sweden under the Tork Brand), see Fig. 1. In the scenario, a brief description of the correct technique of hand hygiene and the five indications for hand hygiene after WHO were presented to the participants. After choosing the profession (physician or nurse), the three virtual patient rooms were entered. In a variety of clinical-practical situations, the participant had to identify the correct indication and sequence of hand hygiene and use of protective gloves. An immediate warning appeared, if a measure was forgotten. After completion of the scenarios, the application directly evaluated the situations and gave feedback by creating an adherence score in percent. Single-use eye masks were handed out to each participant and surfaces of the glasses were disinfected after each use.

Inclusion criteria

- All HCW of the wards under consideration

Exclusion criteria

- The participant suffered from a health condition for which the use of virtual reality would be contraindicated. These included persons with epilepsy, vertigo, psychiatric pathologies, pregnancy, wearers of pacemakers or defibrillators, coronary heart pathologies, migraines, hearing aids, strabismus or amblyopia
- The participant did not follow the instructions or refused to fill out the required documents

### Lecture

A slide show presentation with the correct technique and indications for hand hygiene was conducted.

Inclusion criteria

- All HCW of the wards under consideration

Exclusion criteria

- None

### Outcomes

Primary outcome was the HCW acceptance. For the analysis of acceptance, a Likert scale questionnaire was used with a score of 1–5, where the phrases were "Totally disagree" (1), "Rather disagree" (2), “Neutral” (3), "Rather agree" (4), "Totally agree" (5). The questionnaire was handed out directly after the training in paper form.

Secondary outcomes were hand rub consumption and compliance to indications for hand hygiene as proposed by WHO. Hand rub consumption was assessed by counting the purchase orders during the study period. Compliance observations were conducted before the interventions to create a baseline and after each intervention. They were performed by a research assistant not included in further processes of the trial to guarantee blinding. At least 50 observations of correct hand hygiene according to the five moments of WHO [14] were carried out per ward and period. The outcome was measured approximately two weeks after intervention to possibly exclude acute and only short-lived behavioral adjustments on the one hand and one the other to be able to identify strong enough effects. The above-mentioned procedure resulted in 150 observations for ward 1 and 2 each, 100 observations for ward 3, and 400 observations in total. The observations were carried out according to "Action clean hands", which is a German Health Ministerial supported project based on the WHO campaign "Clean care is safer care" [15]. The data was documented in the software and pre- analyzed in the software “Observe” (Hartmann Group, 2015) using a tablet.

### Data analysis

Chi-square-test and exact-test according to Fisher were carried out for measuring differences in compliance observations before and after the interventions. The hand rub consumption was adjusted to the patient days of the wards. For the assessment of participant’s acceptance to the new training method, a set of 8 items addressing acceptance (3), usability (3), satisfaction (1), and preference (1) with a symmetric and balanced 5-point Likert scale was created [16, 17]. The items were all formulated positively—the higher the score, the higher the acceptance. After, the questionnaires were evaluated by means and standard deviation. Adverse side effects of the VR experience were documented.

## Results

In summary, 81 trainings were conducted, 48 VR trainings and 33 trainings by lecture. At ward 1, 25 HCW participated in the VR training and 13 attended the lecture training. At ward 2, the VR training was conducted in 13 participants and the lecture training was attended by 20. Ten additional HCW participated in the VR training at ward 3.

**Fig. 2:**
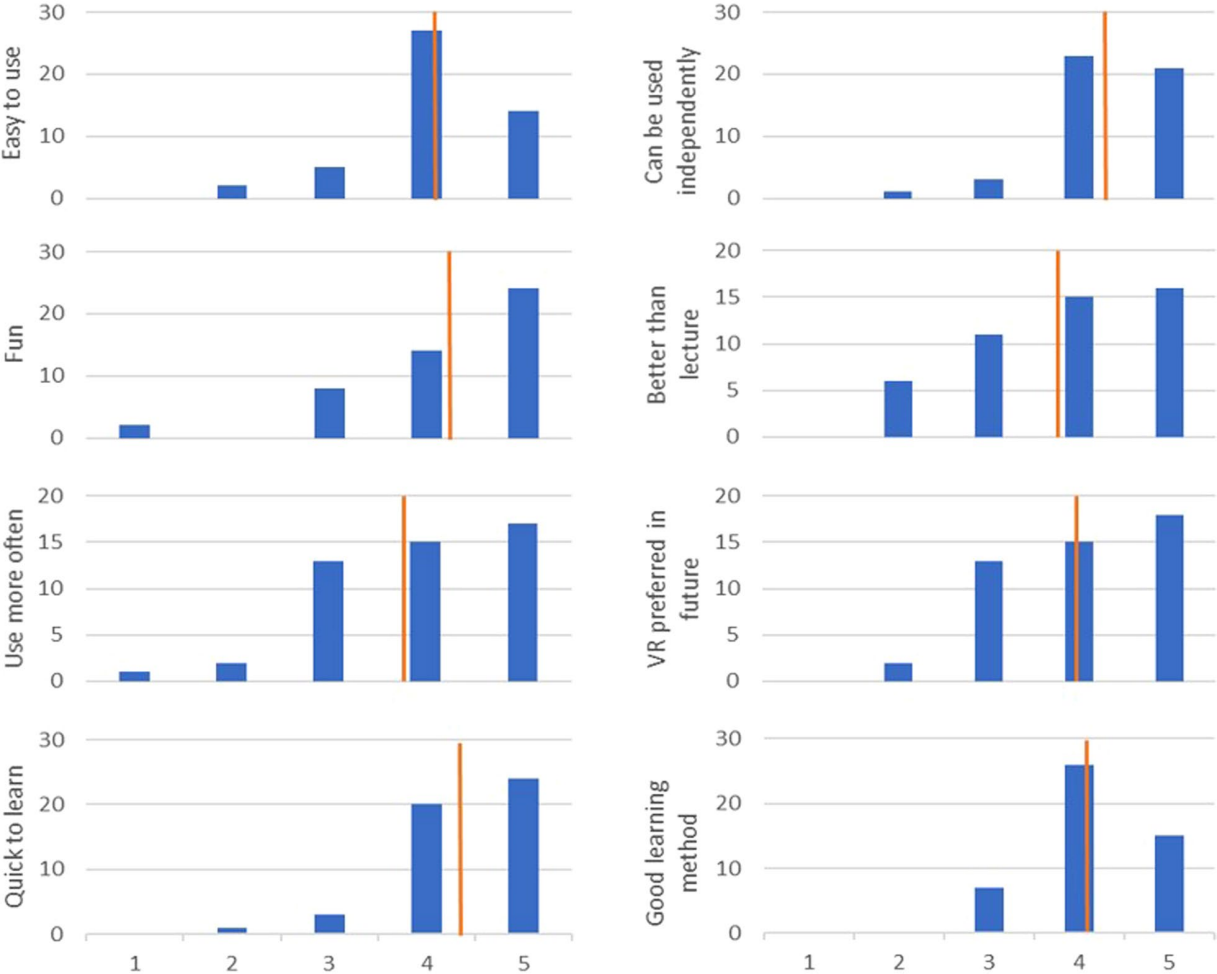
Acceptance of VR training of HCW in 8 items from 1 "Totally disagree" to 5 "Totally agree"; *n* = 48. Number of HCW selecting the grade of acceptance are shown in blue. Mean values are indicated in orange

Overall, VR training was well accepted by HCW with a mean satisfaction in all items above 3, as shown in Fig. 2. In 3 cases, VR training had to be terminated prematurely due to dizziness and/or nausea. The few points of criticism were immature technology and malfunctions, unfamiliar use, and hygiene concerns regarding cleanliness of the devices.

**Fig. 3:**
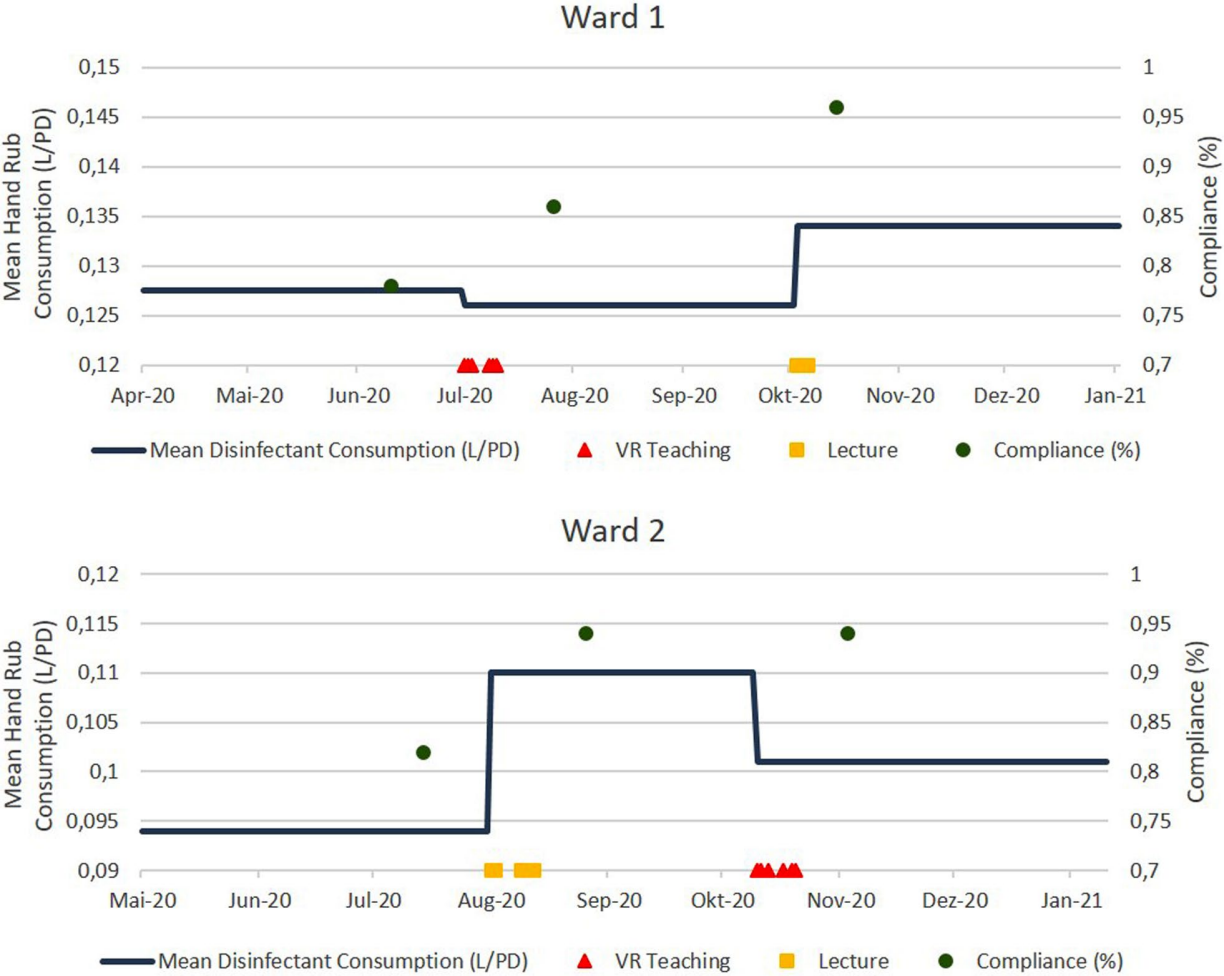
Mean hand rub consumption and compliance of HCW before and after hand hygiene teaching

In total, 400 observations of hand hygiene indications were made, 50 before intervention and 50 after each intervention at the three wards. As shown in Fig. 3, the mean proportion of correct and indication-appropriate performances was 81% before intervention, 87% after VR training, and 95% after lecture. Chi-Square/ Fisher's exact test showed a significant difference after implementation of the lecture compared to no intervention (*p* = 0.04) and a non-significant improvement after VR training (*p* = 0.12). For nursing staff, this result could be reproduced (*p* = 0.009 after lecture and 0.469 after VR training); in physicians, only the corresponding trend was achieved, but not significance (*p* = 0.071 after lecture and 0.067 after VR training). Mean hand rub consumption was 103 mL/Patient-day (PD) in the 2-month period before the first intervention, 90 mL/PD in the 2-month period after VR training, and 125 mL/PD in the 2-month period after the conventional lecture with no significant differences.

## Discussion

To our knowledge, this is one of the first studies that compare Virtual Reality training with conventional lectures for teaching HCWs in hand hygiene. HCWs were extraordinarily satisfied with the new teaching format and gave an encouraging feedback. Most HCW (69%) would prefer VR teaching rather than a lecture for hand hygiene education, while only 4% preferred the traditional lecture. Surprisingly, the observed overall effect on hand hygiene compliance, however, was better after the conventional lecture. A reason for the better compliance after the conventional lecture could be the personal contact to an IPC professional during the lecture that is lacking in the VR training. The possibility to ask questions about knowledge gaps and have them directly answered by the trainer might impact the compliance rate after the training. In the conventional training setting, also questions can be answered that are not covered by the provided training material, while the VR is limited to a pre-programmed scenario which makes it less flexible and adaptable.

Additionally, the lecture is mainly a presentation of hand hygiene indications, while the VR scenarios are configured as a test with feedback, that should enable the transfer of knowledge into practice in a safe environment. Therefore, if the reason for low compliance of HCW was mainly the lack of knowledge of hand hygiene indications, the lecture might have had the better focus. Nevertheless, an explanation of indications could be added to the VR training as well.

Finally, mean compliance with hand hygiene increased after both, VR and conventional lecture-based training.

Researchers of a recent review on existing hand hygiene apps found that most apps do not sufficiently meet quality criteria and concluded that the feasibility and effectiveness of hand hygiene apps should be assessed, especially within healthcare settings [18]. A further VR application that has been evaluated in 29 medical students could not produce a significant difference in hand hygiene compliance compared to a control group that received the traditional learning method [19]. Another interesting approach tries to improve hand hygiene compliance by using an VR application that is visualizing microorganism transmissions [20].

Our study has limitations regarding the secondary outcomes. Although we offered several dates for the training, we could not include all HCWs of the wards in all teaching modes, thus effects may be diminished. Further, compliance observations could have been repeated by multiple observers to increase reliability. Hand rub consumption could only be reported as means due to fluctuation of orders.

Due to the high acceptance of VR technology among healthcare workers, it can be considered an interesting addition to conventional lectures for teaching hand hygiene, since no relevant safety concerns were identified. VR training can be easily repeated in multiple short burst trainings and could therefore leverage learning outcomes and impact. However, the hypothesis that a VR teaching has a higher impact on hand rub use and hand hygiene compliance than a conventional lecture cannot be confirmed.

## Conclusions

All in all, VR can be considered as an advanced and affordable technology for possible future hand hygiene education of health care workers. In particular, the VR training was able to inspire the staff with a possible increase of intrinsic motivation to perform hand hygiene and consequently may reduce healthcare-associated infections.

## Data Availability

The datasets used and analysed during the current study are available from the corresponding author on reasonable request.

## Abbreviations

HAI: Healthcare-associated infections
HCWs: Health care workers
IPC: Infection Prevention and Control
PD: Patient-day
VR: Virtual reality
WHO: World Health Organization
